# Anesthésie d’un greffé cardiaque en chirurgie non cardiaque: à propos d’un cas clinique et revue de la littérature

**DOI:** 10.11604/pamj.2016.24.284.9884

**Published:** 2016-07-28

**Authors:** Oumarou Mahamane Mamane Nassirou, Abdelhamid Jaafari, Abdellatif Chlouchi, Mustapha Bensghir, Charki Haimeur

**Affiliations:** 1 Pôle Anesthésie Réanimation, Hôpital Militaire Mohammed V, Université Mohammed V Souissi, Rabat, Maroc

**Keywords:** Transplanté cardiaque, chirurgie non cardiaque, traitement immunosuppresseur, anesthésie, Heart-transplant patient, noncardiac surgery, immunosuppressant treatment, anaesthesia

## Abstract

Le nombre et la durée de survie des patients transplantés cardiaque sont en augmentation. Une partie de ces patients se présentent régulièrement pour une chirurgie générale en dehors de la transplantation cardiaque. L’anesthésie chez ces patients peut être difficile en raison des particularités physiologiques du cœur dénervé et de la gestion du traitement immunosuppresseur avec le risque de rejet et d’infection. Nous discutons la prise en charge anesthésique à travers un cas d’un patient âgé de 60 ans transplanté cardiaque devant subir une chirurgie de cure d’éventration abdominale et une revue de la littérature.

## Abstract (en)

The number and the overall survival rate of heart transplant patients are increasing. Some of these patients undergo general surgery other than heart transplant on a regular basis. Anesthesia may be difficult in these patients because of the physiological characteristics of the denervated heart and of the management of immunosuppressive therapy on the basis of the risk of rejection and infection. Our study aims to discuss the anesthetic management of a 60-year-old heart-transplant patient undergoing abdominal hernia repair surgery and to write a literature review.

## Introduction

Le nombre de greffes cardiaques est en constante augmentation et beaucoup d’entre eux se présentent de plus en plus pour une chirurgie non cardiaque [1]. A l’occasion de vacances, de déplacements privés ou professionnels, loin de leurs centres de suivi, certains patients vont être amenés à consulter dans l’hôpital le plus proche pour une pathologie variée. Les chirurgies les plus fréquentes sont digestives simples et parfois majeures [2]. Les informations concernant les interactions physiologiques et pharmacologiques dans un cœur transplanté dénervé, les effets secondaires du traitement immunosuppresseur, le risque d´infection et de rejet sont essentiels pour l´anesthésiste dans la prise en charge de ces patients [3]. Nous rapportons le cas d’un patient âgé de 60 ans transplanté cardiaque qui devrait subir une cure d’éventration sous anesthésie générale.

## Patient et observation

Il s’agissait d’un patient âgé de 60 ans devant subir une cure d’éventration abdominale avec mise en place d’une plaque. Dans ces antécédents on notait une transplantation cardiaque en novembre 2014 suite à rétrécissement aortique compliqué d’une cardiomyopathie dilatée. Suite à laquelle Il était mis sous traitement immunosuppresseur à base de ciclosporine, d’evérolimus et de corticoïdes, une insuffisance rénale modérée, une bicytopénie (anémie et lymphopénie) pour laquelle il était sous acide folique, une hypertension artérielle sous traitement béta bloquant et ARA II et une dyslipidémie traitée par la statine. La consultation pré anesthésique effectuée trois semaines auparavant trouvait un patient en bon état général avec une bonne tolérance à l’effort. La tension artérielle était à 154/97 mmHg, la fréquence cardiaque à 80 batt/minute. Il n’y avait pas de critère d’intubation difficile. Sur le bilan biologique, on notait une clairance de créatinine à 46,5 ml/min/1,73m^2^, une hémoglobine à 10,4g/dl, une lymphopénie à 1210 éléments et une glycémie à jeun à1,02g/dl. Sur le plan cardiaque l’électrocardiogramme montrait un rythme régulier non sinusal, des extrasystoles ventriculaires, un bloc de branche droit complet avec des troubles de repolarisation secondaires. La radiographie du thorax montrait une silhouette cardiaque de volume normal et de contour régulier. L’échocardiographie trans-thoracique montrait des cavités cardiaques non dilatées normo-kinétiques avec une fréquence d’éjection ventriculaire à 60%, un péricarde sec, sans aucun signe d’hypertension artérielle pulmonaire. Il n’y avait pas d’anomalies valvulaires en dehors d’une insuffisance mitrale grade I. le dosage de la NT-proBNP était à 1280 pg/ml. Le malade était programmé en première position et les consignes de restriction du personnel dans la salle opératoire avec respect stricte de l’asepsie étaient données avec un séjour dans une salle de surveillance post interventionnelle isolée. Le jour de l’intervention le patient avait reçu son traitement immunosuppresseur et antihypertenseur, seul l’ARA II était arrêté pendant 24h. Après admission au bloc opératoire et mise en place du monitorage standard (FC, SPO2, PNI), une dose de 2 mg de midazolam et 2 g de céfazoline étaient administrés. Après une pré oxygénation et un remplissage vasculaire, l’induction de l’anesthésie générale était démarrée par du fentanyl (3µg/kg) et de l’étomidate (0,2mg/kg). Pour la myorésolution, du cisatracurium (0,15mg/kg) était administrée. L’entretien de l’anesthésie était fait par le propofol à objectif de concentration (concentration cible à 2µg/ml) et par du fentanyl en bolus (25µg/). Durant l’intervention qui avait duré 1h30 min, l’état hémodynamique (FC, PNI) et respiratoire restaient stables. L’analgésie post opératoire était assurée par du paracétamol (1g) et du néfopam (20 mg). Après réveil complet, le patient était extubé sur table puis transféré à la salle de surveillance post interventionnelle. Durant les deux heures de surveillance aucun incident n’a été noté et le patient était transféré au service de chirurgie. La reprise du traitement immunosuppresseurs et antihypertenseurs était faite 6 heures après. L’antibiothérapie était continuée pendant 48 heures. L’évolution était bonne et le patient était déclaré sortant après trois jours d’hospitalisation.

## Discussion

Environ 10 à 25% des greffés subissent une chirurgie non spécifique [4]. Le nombre de patients transplantés cardiaques augmente considérablement dans le monde entier avec un taux de survie de 90% et 50% à 1 et 5 ans respectivement [5]. L’évaluation préopératoire de ces patients subissant une chirurgie non cardiaque devrait se baser sur la fonction du greffon, le risque de rejet, le risque infectieux et la fonction des autres organes qui pourraient être altérés en particulier par le traitement immunosuppresseur ou du dysfonctionnement de l´organe transplanté [6]. Pour la greffe cardiaque, une maitrise des conséquences de la transplantation s’avère nécessaire pour une meilleure prise en charge anesthésique. En effet le cœur transplanté est un cœur dénervé [7]. La dénervation atteint les systèmes sympathique et parasympathique. L’absence d’innervation sympathique explique l’absence de réponse aux stimuli mettant en jeu le baroréflexe (intubation, hypovolémie, manœuvre de Valsalva), l’absence de symptomatologie douloureuse en cas d’ischémie, la réponse exagérée aux sympathomimétiques directs (adrénaline noradrénaline) et celle émoussée aux sympathomimétiques indirects (éphédrine, dopamine). La fréquence cardiaque de base est de 90 à 100 battements par minutes du fait de l’absence de tonus vagal. Sur le plan électro-physiologique, le rythme est le plus souvent sinusal avec une conduction auriculo-ventriculaire normale. Si la technique chirurgicale a laissé en place l’oreillette native du receveur, une deuxième onde P pourra être visualisée à l’électrocardiogramme. Les troubles de la conduction sont fréquents et 5% des patients ont besoin d’un pacemaker dans les mois qui suivent la transplantation. L’autorégulation coronaire est conservée. Cependant un certain degré de ré-innervation peut se produire au cours de l’évolution, rendant compte de certaines situations paradoxales (douleurs angineuses, bradycardie sous néostigmine…). La fonction myocardique dépend du mécanisme de Frank-Starling, requérant une pré charge adéquate pour assurer un débit cardiaque correct. La fonction systolique est préservée alors que la fonction diastolique est plus souvent altérée [8]. Au vu de ce qui précède l’évaluation préopératoire joue un rôle primordial chez le transplanté cardiaque. Cette évaluation consiste à évaluer la bonne fonction du greffon cardiaque par l’évaluation de la tolérance à l’effort, la réalisation d’échocardiographie et/ou d’échographie de stress et si nécessaire avoir l’avis de cardiologue. Notre patient consultait trois semaines auparavant chez son médecin traitant où l’échographie cardiaque ne montrait pas des signes de dysfonction cardiaque avec une FE à 64% sans signes de valvulopathie associés. Il faut également chercher les signes de rejet du greffon cardiaque associant l’apparition d’une fièvre, d’une bradycardie, d’oligoanurie, de rétention hydro-sodée ou de vasculopathie. La vasculopathie ou maladie coronaire du greffon est le facteur limitant la survie à long terme après transplantation cardiaque. Elle concerne 25 à 50% des patients cinq ans après l’intervention [9]. Le risque de rejet est élevé dans les trois premiers mois suivant la transplantation cardiaque d’où l’intérêt de retarder toute chirurgie non urgente durant cette période [10, 11]. Chez le patient transplanté, la gestion du traitement immunosuppresseur est primordiale et doit être poursuivi le matin et le soir de l’intervention pour diminuer le risque de rejet. Les médicaments immunosuppresseurs d´usage courant sont la ciclosporine, l’azathioprine, les antilymphocytaire, les anticorps monoclonaux et les corticostéroïdes. Les nouveaux médicaments tels que le tacrolimus, l’évérolimus et le mycophénolatemofétil remplacent respectivement la cyclosporine et l’azathioprine avec moins d’effets secondaires [12].

Les effets secondaires des immunosuppresseurs qui ont un impact direct sur la prise en charge anesthésique sont l´anémie, la leucopénie, la thrombocytopénie, l’hypokaliémie, l’hypomagnésémie, l´hypertension artérielle, le diabète, la neurotoxique, l´insuffisance rénale, l´anaphylaxie et la fièvre. L’éverolimus retarderait également la cicatrisation et doit interrompu temporairement en cas de chirurgie majeure. Notre patient présentait une bi-cytopénie et une insuffisance rénale modérée secondaire à la prise de la ciclosporine. La ciclosporine et le tacrolimus sont métabolisés dans le foie par le système du cytochrome P450. Il existe donc de nombreuses interactions médicamenteuses entre les inhibiteurs de la ciclosporine et des médicaments régulièrement administrés en péri opératoire, avec des risques de surdosage, et donc d’infection, augmentés ou de sous-dosage avec des risques de rejet augmentés [13]. Par ailleurs, l’association avec certains médicaments comme les anti-inflammatoire non stéroïdiens augmente la toxicité rénale de la ciclosporine et du tacrolimus. Pour ces raisons, il est recommandé de monitorer quotidiennement les taux plasmatiques de tacrolimus ou de ciclosporine en périopératoire et d’éviter autant que possible l’usage de anti inflammatoires. Il existe peu d’études sur les interactions entre immunosuppresseurs et agents de l’anesthésie. Ces interactions ont surtout été étudiées avec la ciclosporine. On retiendra que la durée d’action du fentanyl est augmentée chez la souris [14]; que la durée d’action des curares non dépolarisants est allongée [15]; la concentration alvéolaire minimale de l’isoflurane est augmentée, mais l’isoflurane ne modifie pas les taux plasmatiques de ciclosporine chez l’homme de même que le propofol [16, 17]. En revanche, les doses de myorelaxants doivent être augmentées avec l’azathioprine [18]. La prise en charge peropératoire d’un transplanté cardiaque subissant une chirurgie non cardiaque a pour objectif d´éviter une hypotension, une vasodilatation, ou une diminution brutale du retour veineux du fait de l’importance de la précharge pour maintenir le débit cardiaque. La titration des médicaments est donc souhaitable. De même l’anesthésie générale sera préférée à la rachianesthésie. Le recours aux blocs périphériques est à privilégier [6]. La lidocaïne et la ropivacaïne sont les anesthésiques locaux de choix par rapport à la bupivacaïne dont la toxicité cardiaque est connue. Le type de monitorage (invasif ou non invasif) dépend du type de l’intervention chirurgicale, de la technique d´anesthésie et de l´état du patient. Le monitorage hémodynamique non invasif est à privilégier à cause du risque infectieux lors de la mise en place d’une ligne artérielle ou d’une voie veineuse centrale et dans ce cas le respect de l’asepsie doit être strict. Chez notre patient, le monitorage était non invasif en raison de l’état stable du patient, du risque chirurgical cardiaque modéré et d’absence de modification de la volémie au cours de la chirurgie pour éventration. L’intubation trachéale et la laryngoscopie peuvent ne pas produire une réponse sympathique à cause de la dénervation cardiaque [19].

Dans notre cas, l’état hémodynamique restait stable. Il n’existe pas de contre-indication à un agent ou une technique anesthésique et le choix sera guidé par les dysfonctions de chaque organe (insuffisance rénale, hépatique…) et par le type de chirurgie. Dans notre ce cas, le patient présentait une insuffisance rénale, le cis-atracurium était préféré pour la curarisation du fait de son métabolisme par la voie de Hoffman. L’entretien de l’anesthésie par anesthésie intraveineuse à objectif de concentration a permis d’assurer une bonne stabilité hémodynamique. Les anticholinergiques et anticholinestérasiques utilisés pour la décurarisation ont un effet diminué, voir nul. Toutefois, compte-tenu de la possibilité d’une ré-innervation sympathique, l’administration de la prostigmine doit être toujours associée à l’atropine. En effet, plusieurs cas d’arrêt cardiaque ont été décrits en l’absence de cette précaution [20]. L’infection reste la principale cause de décès chez le transplanté [21]. Cette sensibilité aux infections est en général plus élevée dans la première année après la transplantation et après les traitements des rejets. Toutefois, un épisode récent d’infection doit faire retarder une chirurgie réglée fonctionnelle. Il n’y a pas de règle spécifique au patient transplanté en matière d’antibioprophylaxie. Celle-ci suivra les recommandations des sociétés savantes. Une asepsie rigoureuse est nécessaire. Au moindre signe d’infection, il faut faire des prélèvements multiples à la recherche d’infections opportunistes. Il n’y a pas de particularité pour l’analgésie postopératoire. Toutefois on évitera les anti-inflammatoires non stéroïdiens qui augmentent la toxicité rénale de la ciclosporine. La prévention de l’ulcère, de la maladie thromboembolique complète la prise en charge postopératoire. Une alimentation précoce, en dehors des contre-indications, autorisant la reprise des immunosuppresseurs est préconisée.

## Conclusion

La dénervation cardiaque, la sensibilité aux variations hémodynamiques et aux infections, les conséquences des traitements immunosuppresseurs et leur gestion sont les principales implications anesthésiques d’un patient transplanté cardiaque pour une chirurgie non cardiaque.
